# Near Total Resolution of Severe Refractory Chronic Cough With Low‐Dose Sublingual Buprenorphine: Case Report

**DOI:** 10.1155/crps/1043995

**Published:** 2026-08-13

**Authors:** David W. Streem, Jessica Benovic, Akhil Anand

**Affiliations:** ^1^ Department of Psychiatry and Psychology, Cleveland Clinic, Cleveland, Ohio, USA, clevelandclinic.org

**Keywords:** buprenorphine, case report, chronic cough, quality of life

## Abstract

Refractory or unexplained chronic cough (RUCC) is a cough lasting more than 8 weeks that persists despite guidelines‐based evaluation and treatment, either because no cause is identified or because the cough continues after appropriate treatment of identified conditions. It can significantly impair functioning, sleep, and quality of life. Guideline‐supported options remain limited. We report on a case of RUCC that was very effectively treated with low‐dose sublingual buprenorphine. This partial μ‐opioid agonist can offer greater safety and lower misuse liability compared to full opioid agonists or tramadol. Further study of this treatment option is warranted.

## 1. Introduction

Chronic cough is a common and often challenging clinical problem. Although many cases are attributable to identifiable etiologies such as asthma, gastroesophageal reflux, or upper airway conditions, a substantial proportion of adults complete comprehensive evaluation without a specific diagnosis. Refractory chronic cough refers to cough that persists despite treatment of identified contributing conditions, whereas unexplained chronic cough refers to cough for which no cause is found despite thorough evaluation; the two frequently overlap and are together termed refractory or unexplained chronic cough (RUCC). RUCC can significantly impair functioning, sleep, and the overall quality of life [1].

There are no published human studies examining buprenorphine for RUCC. Several pharmacologic characteristics make buprenorphine an appealing candidate compared to tramadol or full‐agonist opioids such as morphine. Buprenorphine is a partial μ‐opioid receptor agonist with a lower risk of overdose, and tramadol carries risks of seizures and serotonin toxicity, particularly in patients taking serotonergic or CYP2D6‐ or CYP3A4‐inhibiting medications [2]. These limitations highlight the need for safer long‐term antitussive options.

Patients with RUCC often exhibit features of sensory neuropathic cough or laryngeal hypersensitivity, yet few well‐tolerated treatments target central cough pathways [3]. Given buprenorphine’s central antitussive potential and comparatively favorable safety profile, it was considered a therapeutic option for a patient with disabling RUCC who had exhausted conventional treatments. The case below outlines her presentation and response.

## 2. Case Report

The patient was an adult female with a history of opioid use disorder in sustained remission since 2021, major depression of mild to moderate severity, and a seizure disorder who developed a severe and disabling form of RUCC. She worked as a clinical research nurse and maintained a high level of professional functioning for many years. Her coughing episodes were abrupt, occurred without identifiable triggers, and were severe enough at times to induce vomiting. At symptom onset, she had been taking gabapentin 600 mg twice daily for approximately 4 years, and increasing the dose did not improve her symptoms.

Her diagnostic evaluation included chest X‐ray, esophagogram, esophagoscopy, pulmonary function testing, modified barium swallow study, contrast computed tomography of the abdomen and pelvis, FeNO/sputum eosinophils, multiple rhinoscopies, purified protein derivative testing, antinuclear antibody, C‐reactive protein, and pertussis antibody testing. All results were unremarkable.

She underwent multiple empiric treatment trials, each either ineffective or providing relief for fewer than 10 days. These included vocal fold injection with botulinum toxin and filler gel, proton pump inhibitor and H2 blocker therapy, oral prednisone, inhaled corticosteroids, dextromethorphan, benzonatate, fexofenadine, cetirizine, and diphenhydramine.

After evaluation by two otolaryngologists and repeated unsuccessful trials of medications and procedures, tramadol was recommended. She and her family were concerned about seizure risk and the potential for misuse given her history of opioid use disorder. For these reasons, a trial of low‐dose sublingual buprenorphine was pursued. She had never taken buprenorphine previously, including during earlier treatment for opioid use disorder.

Buprenorphine produced a marked reduction in cough frequency and severity. A daily dose between 2 and 4 mg resulted in consistent symptom control for approximately 1 year. When mild recurrence occurred, a small dose increase restored control within 1 week. Her cough has remained well controlled for more than 2 years since initiating buprenorphine. She currently rates her cough symptoms as “0” on a 10 point visual analog scale (VAS), with 10 being the most severe.

Institutional Review Board approval was not required for a single case report per the Cleveland Clinic policy. The patient provided informed consent for the off‐label use of buprenorphine and for publication of her clinical course, which complied with the IRB procedures for case reporting.

## 3. Discussion

The presence of cough‐producing stimuli (e.g., irritants or allergens) is detected by airway vagal nerve afferents that relay signals to the primary sensory and cingulate cortices as well as the cuneiform nucleus and the periaqueductal gray matter in the midbrain [4]. Because these pathways include limbic and affective regions, the direction of causation can be primarily respiratory, psychological, or bidirectional. RUCC can be highly disabling, and many patients demonstrate features consistent with sensory neuropathic cough or laryngeal hypersensitivity. These patients often fail standard therapies that target peripheral or inflammatory causes and may require treatments that modulate central cough pathways. Guideline‐supported options [5] remain limited, and although individual randomized trials have suggested benefit from morphine, gabapentinoids, baclofen, or amitriptyline, the evidence [4] has not been considered sufficient for broad clinical recommendation.

To our knowledge, there are no previously published human cases describing the use of buprenorphine for RUCC. Buprenorphine’s pharmacologic properties make it an intriguing potential therapy in patients who have exhausted established approaches [6]. Buprenorphine’s activity at the μ‐opioid receptor, along with its antagonism at the kappa receptor, may modulate central cough pathways involved in sensory neuropathic cough, a mechanism consistent with emerging models of laryngeal hypersensitivity. Preclinical studies have also demonstrated antitussive effects of buprenorphine, supporting its potential impact on central cough pathways [6]. As a partial μ‐opioid receptor agonist, buprenorphine provides central antitussive activity with a lower risk of respiratory depression and overdose than that of full opioid agonists. Its ceiling effect on respiratory suppression offers a potential safety advantage for long‐term use. Compared with tramadol, buprenorphine may also reduce the risk of seizures or serotonin toxicity, particularly in patients taking serotonergic medications or medications that interact with CYP2D6 or CYP3A4 metabolism [2].

In this case, low‐dose sublingual buprenorphine produced a marked and sustained reduction in cough severity and frequency after numerous failed medical and procedural interventions. Symptom control remained stable for more than 2 years, and even brief recurrences responded quickly to minor dose adjustments. The durability of this response suggests a central therapeutic effect that may be relevant for other patients with severe RUCC, especially those with coexisting neurologic or sensory features.

This report has limitations inherent to a single case, including a lack of objective cough frequency monitoring and the inability to determine generalizability to broader patient populations. Neither chest CT scan, cough‐control therapy, nor speech therapy was pursued. Because no objective cough monitor was used, the magnitude of improvement relies on patient report and clinician observation, and the contribution of natural symptom fluctuation cannot be excluded. However, the magnitude and persistence of improvement, along with the patient’s extensive prior treatment history, support the consideration of buprenorphine as a candidate therapy in selected cases. Further study is needed to better define its efficacy, safety, and optimal dosing for chronic cough, as well as to determine which patient populations might benefit most. Future studies should use validated tools such as the Leicester Cough Questionnaire [7] or the Cough Hypersensitivity Questionnaire [8].

## 4. Conclusion

Severe RUCC remains difficult to manage, especially when patients do not respond to guideline‐supported therapies [5]. In this case, low‐dose sublingual buprenorphine produced rapid, durable, and clinically meaningful improvement after numerous unsuccessful interventions. Although these findings are not generalizable, they highlight a potential role for buprenorphine as a centrally acting therapy in carefully selected patients with RUCC. Further controlled studies are needed to evaluate its safety, effectiveness, and broader clinical applicability.

## Funding

The authors have nothing to report.

## Consent

Permission was obtained from the subject, and a release authorizing publication was signed by the subject for this case report.

## Conflicts of Interest

The authors declare no conflicts of interest.

## Data Availability

The data that support the findings of this study are available upon request from the corresponding author. The data are not publicly available due to privacy or ethical restrictions.
